# Hand explorations are determined by the characteristics of the perceptual space of real-world materials from silk to sand

**DOI:** 10.1038/s41598-022-18901-6

**Published:** 2022-08-30

**Authors:** Dicle N. Dövencioǧlu, F. Seyhun Üstün, Katja Doerschner, Knut Drewing

**Affiliations:** 1 Department of Psychology, Middle East Technical University, 06800 Ankara, Turkey; 2 Cognitive and Social Neuroscience, Adolfo Ibanez University, Santiago de Chile, Chile; 3 Department of Experimental Psychology, Justus Liebig University, Giessen, Germany; 4 National Magnetic Resonance Research Center, Bilkent University, Ankara, Turkey

**Keywords:** Psychology, Human behaviour

## Abstract

Perceiving mechanical properties of objects, i.e., how they react to physical forces, is a crucial ability in many aspects of life, from choosing an avocado to picking your clothes. There is, a wide variety of materials that differ substantially in their mechanical properties. For example, both, silk and sand deform and change shape in response to exploration forces, but each does so in very different ways. Studies show that the haptic perceptual space has multiple dimensions corresponding to the physical properties of textures, however in these experiments the range of materials or exploratory movements were restricted. Here we investigate the perceptual dimensionality in a large set of real materials in a free haptic exploration task. Thirty-two participants actively explored deformable and non-deformable materials with their hands and rated them on several attributes. Using the semantic differential technique, video analysis and linear classification, we found four haptic dimensions, each associated with a distinct set of hand and finger movements during active exploration. Taken together our findings suggest that the physical, particularly the mechanical, properties of a material systematically affect how it is explored on a much more fine-grained level than originally thought.

## Introduction

The perceived properties of a material guide critical decisions about how to interact with an object, for example, whether we should eat it, buy it, sit on it or how we should pick it up. How we perceive materials has been a topic of longstanding interest in the haptic domain, yet only recently we begin to understand how the haptic perceptual space for materials might be structured. For example, on the basis of a meta-analysis of 18 studies, Okamoto et al.^1^ characterized this space to vary along five main factors: macro and fine roughness, warmness (cold-warm), hardness (hard-soft), and friction (moistness-dryness, stickiness-slipperiness). More recently, using a much larger set of materials (120), these five factors have been replicated and extended by two (volume and naturalness) by Sakamoto and Watanabe^2^. However, in these studies the actual exploration of the materials has been restricted to passive, or single digit active lateral or indentation movements. Yet, when we naturally interact with the materials around us, we typically engage the entire hand and all digits and perform much more complex movements than lateral motion or pressing down.

In haptic object perception we know that animals, including humans, adapt their exploratory hand movements while perceiving 3D shapes, in order to stimulate the appropriate skin receptors^3^. Such stereotypical hand movement patterns in active touch have been coined exploratory procedures (EPs) by Lederman and Klatzky^4^, and they are thought to be optimal for perceiving a particular object property, e.g. its shape and texture, but also its material properties such as temperature or softness. When hand motion is restricted^1,2^ there is a risk that the perceiver has only limited access to the material property needed to make a perceptual judgement about a material quality. This, in turn, might affect how the perceptual space obtained from these experiments is structured. Our own informal observations showed that under natural exploration conditions observers use a great variety of hand movements during interactions with materials (e.g. in Drewing et al.^5^). This might be particularly pronounced when exploring nonrigid materials: we explore fur or velvet with stroking motion, we tend to run our fingers through fine sand, and we press them into playdough. Velvet, beach sand and playdough are all nonrigid materials but they differ substantially in their mechanical properties, i.e. how they deform in reaction to force, and we seem to interact with each one of them in idiosyncratic ways, that seems to be modulated primarily by those mechanical properties (e.g. as opposed to temperature). Given this, in the present study we focus on the perception and exploration of mechanical material properties, with a two-folded goal: (1) to test the dimensional architecture of the haptic perceptual space for mechanical material properties when participants explore samples unrestricted and (2) to assess whether the obtained perceptual dimensions are associated with distinct patterns of exploratory hand movements^2,3^.

Using a free manual exploration in conjunction with a rating paradigm and video analysis we find, consistent with previous work, that there are several perceptual dimensions that represent mechanical material properties, several of them related to the construct of “softness”. In addition, we show that each perceptual dimension is associated with a different set of EPs, and that these EPs are much more fine-grained than those described by Lederman and Klatzky^4^.

## Results

### Ratings

#### Consistency

First we wanted to know whether different participants judged the materials in similar ways. In order to assess interindividual consistency of the ratings, we calculated correlations and Cronbach’s alpha (formulated from the number of adjectives, covariance, and variance in the data). The pairwise correlations for all materials and all adjectives between all participants were on average r = 0.58. Cronbach’s alpha was in the range of 0.9–0.99 across the set of adjectives, suggesting, high consistency among participant’s ratings.

#### Exploratory factor analysis

To explore the patterns in the adjective ratings, we used a factor analysis that gathers similar responses together (in particular adjectives with highly correlated ratings) in a factor and represents the whole dataset with less dimensions. Since we did not know how many factors this dataset would reveal, we used an exploratory factor analysis. Before the analysis, we wanted to see the variance adequacy of our sample with a KMO test and whether the variances for each item (adjective rating) are equal with the Bartlett scores. The KMO score was .703 and Barlett’s test of sphericity was significant ($\chi ^2(465) = 2574.59, p<0.0001$), suggesting that the data were appropriate to conduct a Factor Analysis and that the overall significance of all correlations within the matrix was meaningful (and correlations were not just random). Applying the Kaiser-criterion, the factor analysis revealed five factors in the data, explaining the 87.26% of the variance in total. Table 1 shows factor loadings after varimax rotation. In order to obtain a criterion value to determine relevant factor loadings, communalities of adjectives (ranging from min = 0.36 to max = 3.66 with mean = 2.46) were used. The square root of 30% of the mean communality is obtained as a criterion score (c = 0.736). Each adjective above this cut-off value is highlighted with italics in the Table 1. Adjectives, highlighted in bold have maximal loadings across factors.

**Table 1:** List of 31 adjectives in English and their Turkish translations with Factor loadings after varimax rotation.

Adjective in English	Turkish translation	Compliance	Viscosity	Surface softness	Granularity	Roughness
Elastic	Elastik	**1.508**	0.120	0.117	$-0.462$	0.024
Inflexible	Esnemez	$-\textbf{1.493}$	$-0.226$	$-0.394$	0.290	$-0.191$
Compliant	Güç uygulanabilir	**0.843**	0.060	$-0.056$	$-0.305$	$-0.053$
Malleable	Biçimlenebilir	**0.985**	0.378	0.080	$-0.073$	$-0.095$
Hard	Sert	$-\textbf{1.391}$	$-0.681$	$-1.056$	$-0.032$	$-0.191$
Soft	Yumuşak	**1.355**	*0.814*	*0.969*	$-0.203$	0.111
Delicate	Hassas	**0.762**	0.183	0.497	$-0.099$	$-0.086$
Doughy	Hamursu	**0.924**	*0.764*	$-0.180$	$-0.119$	$-0.177$
Meaty	Et gibi	0.384	0.176	$-0.162$	$-0.131$	$-0.062$
Spongy	Süngerimsi	**0.783**	$-0.070$	0.388	$-0.237$	0.721
Woody	Odunsu	$-0.612$	$-0.254$	$-0.519$	0.003	0.255
Fluffy	Kabarık	0.669	$-0.068$	0.300	$-0.111$	0.529
Sticky	YapıŞkan	0.206	**1.408**	$-0.087$	0.017	$-0.273$
Gooey	Vıcık vıcık	0.363	**1.552**	$-0.101$	$-0.059$	$-0.296$
Slimy	Sümüksü	0.336	**1.271**	$-0.143$	$-0.112$	$-0.250$
Moistrous	Nemli	0.119	**1.420**	$-0.019$	$-0.094$	$-0.181$
Gelatinous	Jölemsi	0.534	**1.290**	$-0.176$	$-0.135$	$-0.311$
Silky	Ipeksi	0.146	$-0.057$	**0.923**	$-0.119$	$-0.062$
Velvety	Kadifemsi	0.115	$-0.165$	**1.112**	$-0.277$	0.451
Hairy	Tüylü	0.063	$-0.154$	**0.961**	$-0.218$	0.428
Airy	Tiril tiril	0.050	$-0.124$	0.530	0.139	$-0.075$
Scabby	Kabuklu	$-0.320$	$-0.191$	$-0.375$	0.215	0.303
Sandy	Kum gibi	$-0.309$	$-0.050$	$-0.025$	**1.548**	0.052
Powdery	Toz gibi	$-0.220$	0.087	0.081	**1.465**	0.003
Granular	Tanecikli	$-0.457$	$-0.120$	$-0.305$	**1.747**	0.330
Scaly	Pul pul	$-0.227$	$-0.206$	$-0.245$	**0.782**	0.361
Leathery	Derimsi	0.277	$-0.079$	0.283	$-0.296$	$-0.008$
Textured	Dokulu	$-0.133$	$-0.293$	0.339	$-0.015$	**0.879**
Roughened	Pürüzlü	$-0.413$	$-0.376$	$-0.053$	0.554	**1.142**
Slippery	Kaygan	$-0.097$	0.704	0.253	$-0.214$	$-\textbf{0.796}$
Glossy	Parlak	$-0.141$	0.187	0.022	$-0.201$	$-\textbf{0.764}$

The first rotated factor explains 25.68% of variance in data. The adjectives, “elastic”, “compliant”, “delicate”, “soft”, “spongy”, “malleable”, “doughy” (positive loadings) and “inflexible”, “hard” (negative loadings) load highly in this factor. Taken together, the elasticity (elastic, compliant, doughy delicate, and inflexible, hard in reverse meaning) of the material might explain this factor loading. Furthermore, for negative loadings, non-compliant seems to contribute to the loadings of the adjectives inflexible and hard. Thus, we labeled this factor compliance. The second factor explains 22.62% of variance. Adjectives “moisturous”, “gooey”, “sticky”, “slimy” and “gelatinous” load high on this factor. Slipperiness, wetness and density features of materials seem to play a combined role in the loading of this factor. Therefore, this factor was labeled viscosity. The third factor explains 12.44% of variance. Adjectives “velvety”, “silky” and “hairy” load on this factor. This factor was labeled surface softness. The fourth factor explains 16.92% of variance. “Sandy”, “powdery”, “granular” load very highly on this factor, along with a relatively lower loading of “scaly”. We suggest that material properties which explain this factor loading are the those related to the size and weight of individual material particles: thus, this factor was labeled as granularity. The fifth factor explains 9.6% of variance. “Roughened”, “textured” load high on this factor along with “slippery” and “glossy” (negative loadings). Therefore, this factor was labelled as surface roughness.

### Video analysis

#### Event coding

**Figure 1:**
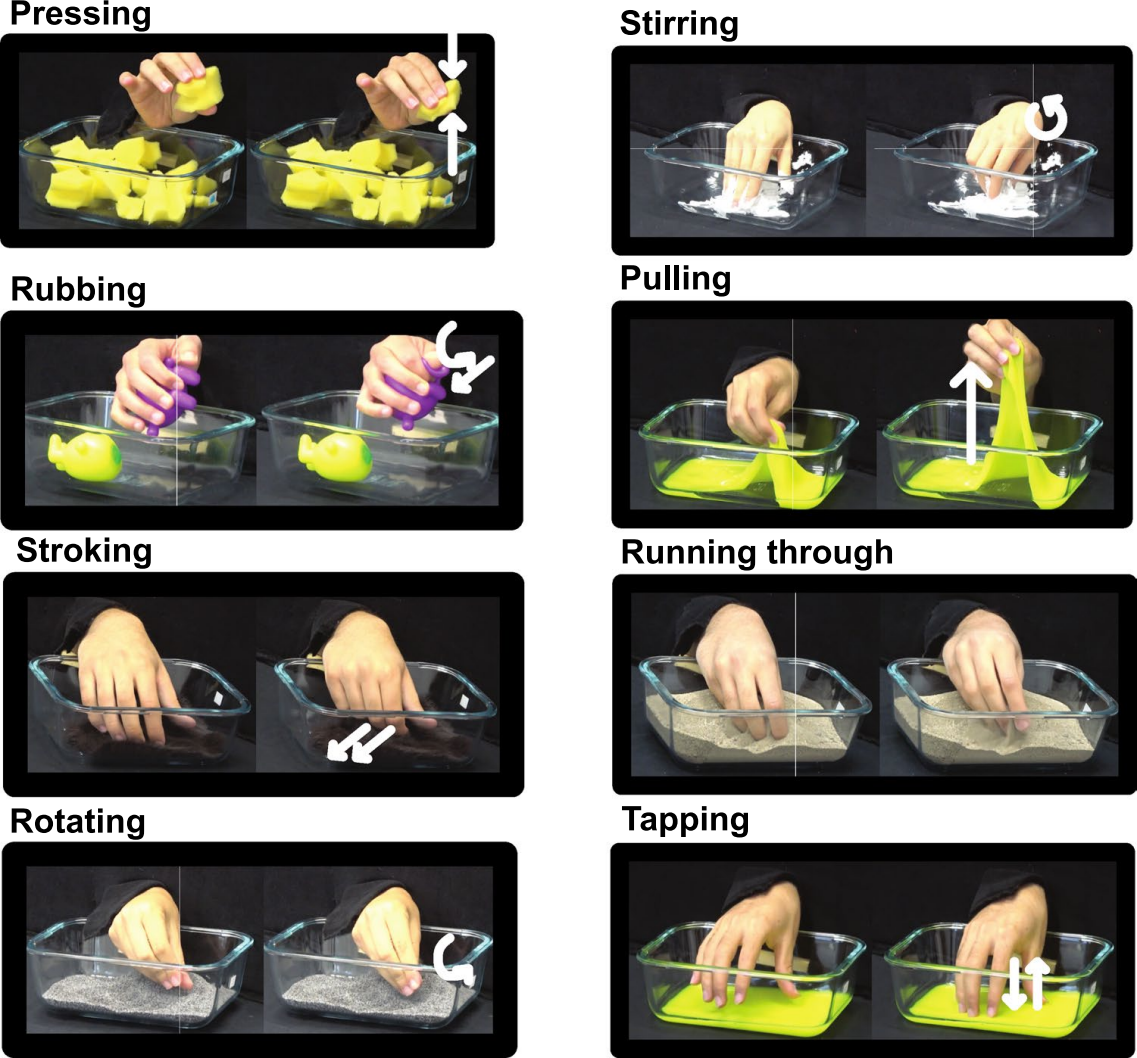
Screenshots illustrating the 8 EPs proposed in this study. Arrows show the direction of hand movements: pressing sponge pieces, rubbing stress balls, stroking fur, rotating poppy seeds, stirring hand cream, pulling slime, running the fingers through sand, and tapping fingertips onto slime.

From our video data we extracted a new set of EPs with the following taxonomy. Examples are shown in Fig. 1.

**Pressing** Applying directional normal force to the material at hand either by squeezing the material between fingers and palm, or applying force to a part of the material with one or more fingers. This would also be categorized as pressure by Lederman and Klatzky^4^.

**Rubbing** Applying torque and lateral force on the materials, sometimes by sweeping thumb and index finger tips against each other, or by forcefully stroking the material with the thumb while using other four digits to stabilize the material. Regardless of the material being picked up or not, rubbing here is defined by the force and torque of the fingers. This EP can be applied in multiple directions.

**Stroking** Moving the hand gently against the material surface. Most frequently the inside of the hand is used to stroke but we encounter stroking with the back of the hand as well (e.g. fur). If it is with the thumb, or as strong as to deform the object then it is considered to be rubbing (Stroking would be categorized as lateral motion in Lederman and Klatzky^4^).

**Rotating** Lifting portions of the material to move and turn its boundary inside the fingers or fingertips.

**Stirring** Moving one or more fingers immersed in the material, sometimes rotationally.

**Pulling** Stretching a part of the material by moving the hand away, or separating the fingers from inside for sticky viscous materials (e.g., hand cream) or outside the fingers in case of extensile materials, e.g., rubber bands.

**Running through** Lifting parts/portions of the material and letting them fall through the fingers. This might also be combing through the material, observable also in hairy or viscous materials, e.g., fur or hand cream.

**Tapping** Hitting the fingertips, knuckles, or the back hand lightly and repeatedly against the material, more rapidly but lighter than pressing. This EP would provide similar kinesthetic cues to that defined in Friedman, Hester, Green, and LaMotte^6^ but since here we do not use a stylus, skin cues would also be available to the participant. This EP might also include lifting the object and hitting it lightly against the container walls.

#### Inter-rater reliability

Figure 4, right, shows correlation coefficients between raters averaged across materials, and across participants all of which are significant at $p < 0.0001$ (for 10 pairs of raters $\times$ 50 videos = 500 comparisons) with Pearson correlation coefficients of 10 averages ranging from $R = 0.70$ to $R = 0.95$. This suggests that raters’ EP assessments had high degree of agreement, and that our proposed EPs and their descriptions provide a valid measure for evaluating exploratory hand movements. In order to check whether the interrater reliability also holds at a more fine-grained level we also computed correlations across timeslots of 30 second bins, i.e., one correlation per rater pair across 10 materials $\times$ 5 subjects $\times$ 4 timeslots. These correlations were still acceptable, with an average around $R = 0.51$. In all following analyses, we will evaluate EP frequencies over the whole time series.

#### EP frequency

**Figure 2:**
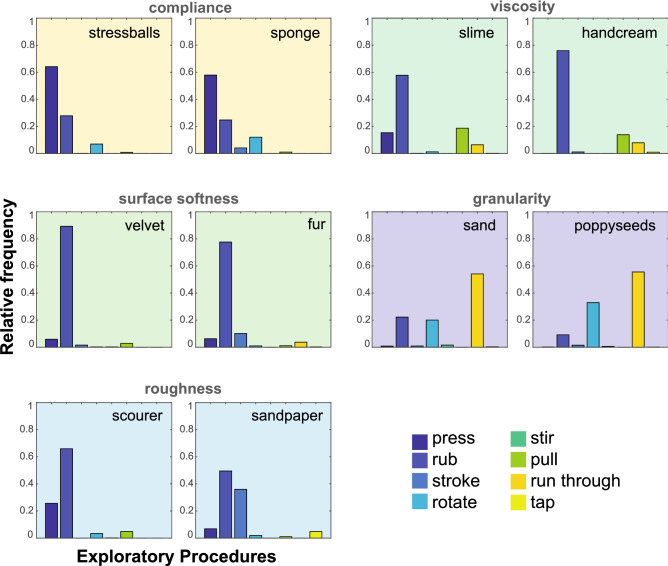
Plots of EP frequencies for each of the 10 materials selected for this analysis. This representation disregards the order of EPs. Each pair of materials is representative of a particular perceptual dimension: four dimensions are related to mechanical properties and one related to roughness.

Figure 2 illustrates that participants tended to employ the same EPs for materials that yielded high values in the same perceptual dimension (e.g., stress balls and sponge on compliance). For compliant materials, the most frequent EP was pressing. For viscous, soft surfaced, and rough materials the most frequent EP was rubbing. For granular materials such as poppy seeds and sand, most of the time people ran their fingers through the material.

EP frequencies obtained from the coding of 50 videos (10 per perceptual dimensions consisting of two exemplary materials each) were averaged across 5 raters. We conducted a one-way MANOVA, where the frequencies of the 8 EPs were the dependent variables and the fixed factor was the material’s dominant perceptual dimension (1–5). We used a 0.05 cut off for significant p values. For each perceptual dimension 10 observations were entered (5 participants $\times$ 2 materials). The analysis revealed a significant main effect of the perceptual dimension ($F(28, 142) = 12.67, p < 0.001$, $Wilk's\;\Lambda = 0.011$, $partial \; \eta ^2 = 0.68$), suggesting that the distribution of frequencies of the 8 EPs depended significantly on the materials’ dominant perceptual dimension. Subsequent univariate ANOVAs revealed that the main effect of perceptual dimension was significant for all EPs except for stir and tap (pressing: $F(4, 45) = 30.33, p < 0.001$, $partial \; \eta ^2 = 0.73$; rubbing: $F(4, 45) = 19.35, p < 0.001$, $partial \; \eta ^2 = 0.63$: stroke: $F(4, 45) = 4.41, p = 0.004$, $partial \; \eta ^2 = 0.28$; rotating: $F(4, 45) = 13.14, p < .001$, $partial \; \eta ^2 = 0.54$; pull: $F(4, 45) = 4.56, p = 0.004$, $partial \; \eta ^2 = 0.29$; running through: $F(4, 45) = 25.27, p < 0.001$, $partial \; \eta ^2 = 0.69$; stir: $F(4, 45) = 1.47, p = 0.23$, $partial \; \eta ^2 = 0.12$; tap: $F(4, 45) =0.94, p = 0.45$, $partial \; \eta ^2 = 0.08$ with a Bonferroni corrected alpha value of 0.00625).

We then wanted to understand the specific differences in EP patterns across the perceptual dimensions. To this end, we compared each EP’s frequency between any two perceptual dimensions using Tukey’s HSD post-hoc test. This yielded several significant differences, which we report next:

Pressing was most frequently observed for compliant materials, significantly more than for materials representing the viscosity, surface softness, or granularity dimensions ($\Delta m_{compliant{\text{-}}granular} = 60.8$, $\Delta m_{compliant{\text{-}}viscous} = 52.6$, $\Delta m_{compliant{\text{-}}surface\;soft} = 54.8$, $\Delta m_{compliant{\text{-}}surface\;rough} = 45.5$, each $p < 0.001$, where $\Delta m$ indicates the percent mean difference in EP frequency with respect to the subscripted perceptual dimensions).

Rotating was most frequently observed for materials representing the granular dimension, ($\Delta m_{granular{\text{-}}compliant} = 17.8$, $\Delta m_{granular{\text{-}}viscous} = 27.0$, $\Delta m_{granular{\text{-}}surface\;soft} = 27.0$, $\Delta m_{granular{\text{-}}surface\;rough} = 25.2$, each $p < 0.003$).

Pulling was most frequently used for viscous materials. The percent mean difference in EP frequency was statistically significant between viscous and granular, compliant and soft surface materials ($\Delta m_{viscous{\text{-}}granular} = 14.7$; $\Delta m_{viscous{\text{-}}compliant} = 13.7$; $\Delta m_{viscous{\text{-}}surface\;soft} = 12.8$; $\Delta m_{viscous{\text{-}}surface\;rough} = 12.3$, each $p < 0.029$).

Running through was most frequently observed for materials representing the granular dimension, ($\Delta m_{granular{\text{-}}compliant} = 50.6$, $\Delta m_{granular{\text{-}}viscous} = 43.8$, $\Delta m_{granular{\text{-}}surface\;soft} = 53.0$, $\Delta m_{granular{\text{-}}surface\;rough} = 49.2$, maximum $p < 0.001$).

The average rubbing frequency for compliant materials was lower than for any other perceptual dimension ($\Delta m_{compliant{\text{-}}viscous} = -40$, $\Delta m_{compliant{\text{-}}surface\;soft} = -57.9$, $\Delta m_{compliant{\text{-}}surface\;rough} = -33$, p < 0.001) except for granularity ($\Delta m_{compliant{\text{-}}granular} = -6.8$, $p = 0.94$).

Overall, stroking occurred most frequently for materials representing the surface roughness dimension. The percent mean frequencies of stroking in this dimension were significantly higher than that for compliance, granularity and viscosity ($\Delta m_{roughness-compliant} = 16.1$, $\Delta m_{roughness-granular} = 16.8$, $\Delta m_{roughness-viscous} = 16$, each $p < 0.013$), but not different from percent mean frequencies of stroking materials that represent roughness and surface softness ($\Delta m_{roughness-surface soft} = 12.3$, $p = 0.088$).

All other remaining comparisons including the percent mean frequencies of stirring and tapping did not reveal any significant differences between the perceptual dimensions.

In general, rubbing was the only EP that was coded for nearly all raters used in nearly all video clips. It was also the EP with the longest duration (ratio to overall duration, mean of five raters = 49.3%), followed by pressing (22.2%), and running through (11.2%). The total duration for the remaining five EPs constitutes 17.2% percent of the total time. Rubbing has previously been described as a prolonged event, compared, for example, to brief events such as pressing or tap^3^. Our results are consistent with this observation. Overall, we find that there are EPs that appear to be paired specifically with one perceptual dimension: participants tend to primarily use pressing for exploring compliant materials, and they tend to use running through and rotating in order to explore granular materials.

In order to validate these results, we then increased the number of video sets to be rated to 100 (10 materials $\times$ 10 participants). One of the 5 raters performed the EP coding of this extended set. When we repeat the MANOVA on the mean EP frequencies on this larger data set we find very similar results to the analysis described here. We subsequently used the EP frequencies obtained from these 100 videos as the data set for the classification analysis reported next.

### Classification

**Figure 3:**
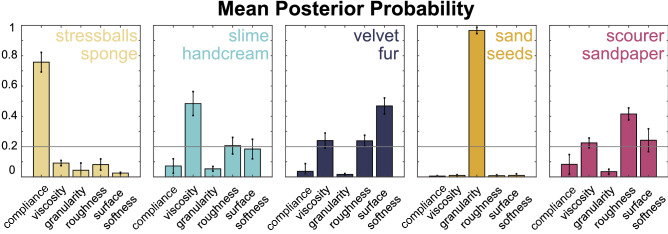
We show the performance of a support vector machine predicting the perceptual dimension from EP frequencies. The algorithm was trained on relative EP frequencies in order to predict the perceptual dimension that a new EP pattern is likely to belong to. Since the classifier was trained to discriminate between five classes, the chance performance level was at 20 per cent (horizontal gray line). For each tested EP frequency type (where type refers to the perceptual dimension that the explored materials scored high on) we also plot the confusions that the classifier generated.

We wanted to test whether EP frequencies for a given material could predict the perceptual dimension that this material most likely represented. A support vector machine (SVM) was iteratively trained to discriminate between the five 5 perceptual dimensions using corresponding EP patterns. We trained the model with 100 observations for 10 materials. Then we used this dataset (100 observations) in a Matlab crossval function with its default variables which translate to 10-fold cross validation by leaving 10 percent of the data out for each iteration. The function randomly picks 10 observations for test and trains the model with the remaining 90 observartions. The goal was to predict, for a given EP pattern (obtained from exploring a particular material), the perceptual dimension that this material is likely to belong to. Fig. 3 shows that the classification of perceptual dimension on the basis of EP patterns was very successful, with the best performance for predicting the granularity dimension (96.6%) from the EP patterns, followed by predicting compliance (75.7%), viscosity (48.4%), surface softness (46.8%), and roughness (41.6%). Each panel in Fig. 3 shows the corresponding confusion errors for a given EP pattern, organized by perceptual dimension that the materials—from which test EPs were obtained—represent. Table 2 shows average values and standard deviations for the corresponding data.

**Table 2:** Mean posterior probabilities and standard deviations from the classification analysis as shown in Fig. 3.

	Compliance	Viscosity	Granularity	Roughness	Surface softness
Stressballs, sponge	$0.76 \pm 0.07$	$0.09 \pm 0.02$	$0.04 \pm 0.05$	$0.08 \pm 0.04$	$0.03 \pm 0.00$
Slime, handcream	$0.07 \pm 0.05$	$0.48 \pm 0.08$	$0.05 \pm 0.02$	$0.21 \pm 0.06$	$0.18 \pm 0.06$
Velvet, fur	$0.04 \pm 0.05$	$0.24 \pm 0.05$	$0.02 \pm 0.01$	$0.24 \pm 0.04$	$0.47 \pm 0.05$
Sand, poppy seeds	$0.01 \pm 0.00$	$0.01 \pm 0.00$	$0.97 \pm 0.02$	$0.01 \pm 0.01$	$0.01 \pm 0.01$
Scourer, sandpaper	$0.08 \pm 0.07$	$0.22 \pm 0.03$	$0.03 \pm 0.02$	$0.42 \pm 0.04$	$0.24 \pm 0.08$

## Discussion

Mechanical material properties cover a broad variety of features that can be related to the texture of food, as much as to the feel of a velvet cloth or the properties of the ground that we step on. Haptic research on materials, however, has focused often only on 2D textures with a few and limited exceptions of compliant stimuli that explicitly vary in mechanical properties such as springs or silicone-like objects. Here, we moved beyond the classic set of stimuli used in haptic material research and included a wide range of real, deformable and non-deformable materials, which we selected to cover a broad variety of materials that are expected to change shape through interaction. A few recent studies, that also included a more diverse set of simulated or real deformable materials (e.g., viscous materials or fabrics), found that, deformable materials indeed afford more than just a percept of elasticity and included also a percept of firmness^7^ and surface softness^8^. To our knowledge, here we have included granular materials to our stimulus set for the first time. Admittedly, these materials (along with our stimulus set) differ in further physical characteristics such as shape, weight, and thermal conductance; but since our set of adjectives were knowingly selected to cover compressibility and compliance characteristics of softness, our findings predominantly concern the perceptual dimensions that are related to the mechanical properties of the material. By using the semantic differential technique, we obtained a total of four perceived dimensions that link to mechanical properties: compliance, viscosity, surface softness and granularity. Strikingly, each of these perceptual dimensions was also associated with a specific set of exploratory procedures, where six of the eight EPs that we found to be relevant for the exploration of deformable (and non-deformable) materials, were previously unknown. In fact, we were able to use the frequencies of these eight EPs for a given material to successfully predict the perceptual dimension that the material was most likely to be associated with. Our findings may also be taken to discuss the prevalent notion of softness. Traditionally, haptic softness has been equated with the compliance of elastic materials or springs, degree of softness measured as the degree of compliance of elastic materials^8–11^, where compliance has been defined as an object’s deformation under force. Di Luca^12^ described haptic softness more generally, as the subjective impression of the compressibility and compliance characteristics of things and materials. Indeed, materials used in this study were all deformable, but how these materials deform differs quite a bit: for example, elastic rubber balls bounce back to their original geometry after compression, whereas clay keeps its deformed shape after the compressing force is removed. Sand, slime, velvet or a rabbit’s fur also deform, but in very different ways than rubber and clay. All of these materials would fall under Di Luca’s^12^ definition of ‘soft’, and also our everyday experience would attribute them some aspect of softness; sand, clay, cream or fur can all be called soft. In that way, one may speculate that our four perceptual dimensions that link to mechanical properties could represent different softness dimensions (with different associated EPs). However, for the present naturalistic stimuli, it is hardly possible to completely isolate the perceptual contribution of softness from that of other sensory dimensions, and future research is required to test this novel notion of a multi-dimensional softness.

It has been proposed that exploratory procedures optimize the gathering of sensory information for the purpose or task to which they are linked^3,4^. For example, in order to gain knowledge about the exact shape of an object, it might be the optimal strategy to follow its contour, or in order to learn about the same object’s weight, it would be best to lift the object away from any supporting surface^4^. Thus, the task strongly determines which EP is going to be predominantly used. While we vary the task in our study, i.e., the adjectives that the material has to be rated on, it always remains—a global level—related to the substance properties of the stimuli. Therefore, one might expect that the four EPs, that Lederman and Klatzky^4^ proposed to be related to the substance—or material—of an object (lateral motion, pressure, static contact, unsupported weight) might suffice to perform this task. However, this is not what we found.

Instead we found, that EPs vary systematically, depending on which material is explored, which implies that they may also be optimized with respect to the material properties, i.e., the specific (mechanic) characteristics (granularity, compliance, etc.), of the to-be-explored object. However, there was not a perfect one-to-one mapping between EPs and the perceptual characteristics. For example, rubbing was the most frequent EP for two of the four perceptual dimensions (surface softness, viscosity), and consequently, rubbing was observed as much for velvet and fur as it was for slime and hand cream. Also, for rough (non-deformable) materials rubbing was the most frequently observed EP. What might explain this prevalence of rubbing as a particularly informative EP in that it provides both vibration and pressure information to the mechanoreceptors which would otherwise be missing with other EPs, such as pressure or stroking (duplex theory^13,14^).

While for some soft surface materials, like silk, rubbing clearly dominated the EP patterns; for other materials, like viscous ones, it was often supplemented by pulling and running through. This suggests, that information gathered by rubbing alone might well be sufficient for the judging surface softness of textiles, but that for judgments of viscosity additional sensory information is required. For example, by pulling the fingers apart, participants assess stickiness of the material, which is related to the viscosity.

Several types of sensory information (and thus several EPs) were apparently also needed for judging granularity, for which we found only a low occurrence of rubbing. Instead, rotating and running through were the most prominent EPs for granular materials, such as sand and poppy seeds. Here, rotating might provide information about the individual grain size and shape, and running through might help estimating other characteristics of the grains by stimulating Meissner receptors, which are known to be good at detecting change and speed. Overall, it appears that the EP’s we discovered here are—as much as the â€˜classic’ ones—optimized. What is new in our study is the discovery that the selection of EP’s can be fine-tuned to the properties of the to-be-explored materials. Specifically, materials that score similarly on a specific perceptual dimension were also explored by similar hand motions.

How then, do our EP’s compare specifically to those proposed by Lederman and Klatzky^4^? As the authors, we also propose an EP called pressing. Pressing occurred more frequently for compliant materials such as sponge and stress balls, together with rubbing and rotation. However, pressure as defined by Lederman and Klatzky^4^ would not be synonymous with our pressing. It would also comprise in part our EP descriptions of rubbing or tapping—depending on the visible effects of force and torque. Similarly, lateral motion^4,13^, would not just map directly onto one of our proposed EPs but instead be coded as either stroking or rubbing—depending on the observed degree of force applied to the material. Interestingly we found that stroking, was related to both, the perceptual dimension of surface softness, as well as to roughness, although it is possible that these two types of stroking might differ in ways that we did not capture here. While some of the differences between Lederman and Klatzky^4^ and our material related EPs are clearly attributable to our more diverse set of materials, some might be also related to the fact that we did differentiate more finely between EPs.

Are really this many distinct EPs needed to describe the manual exploratory interactions with materials? As described above, previous research has nearly exclusively associated pressure with the perception of deformable, in particular compliant materials. However, our results indicate that deformable materials have more than just one perceptual dimension. Thus, it appears plausible that different ‘tools’ (sensory input) are needed to gather information about these dimensions. The successful classification of EP patterns as belonging to one of the proposed perceptual dimensions was only possible because there are distinct EP patterns associated with each perceptual dimension: people apply pressure when judging the properties of deformable materials, let grains and sand run through their fingers, or pull viscous materials. It is very unlikely that pressing alone would suffice to explore all of these perceptual dimensions of soft materials. Nevertheless, this would be an interesting question for further study, namely, to test whether by forcing participants to use non-optimal EPs, percepts of material properties might be altered by the hand movements.

One may think that not all EPs can be applied to all the materials, and this may have led to overestimation of material-specific preferences. Admittedly, it is more difficult to apply some EPs to some materials than others. Although we argue that it is not impossible, it may be counterintuitive. For example, one might think it is not possible to pull sand, but by our definition pulling also includes separating fingers with material smudged, such as checking for stickiness of cream. Hence, it is, in principle, possible to perform pulling with granular materials. Similarly, we define run through including a combing like sweeping motion, which is intuitive while petting a dog, but also not impossible to apply to the surface of a non-granular, non-hairy material such as slime. Having said that, we would like to acknowledge that for a specific material not choosing certain EPs in a few selected cases, also might be due to kinematic difficulties with the material. But does that mean that “true” preferences for an EP are overestimated? This argument would make sense if there was a finite fully described number of potential EPs that are possible at all, and further limitations of the EPs by the material would yield to an overestimation of the usage of the residual EPs in the set. However, human movement control is quite flexible and adaptive, a manifold of different EPs could have been observed, and the question which of theoretically possible EPs are actually used was also part of this study. That is, the baseline for discussing the preferences is not the limited number of 8 observed EPs, but the (infinite) number of theoretically possible EPs. Hence, it would not change much in the interpretation if a few EPs were not possible. We would also like to note that before the experiment we do not know which exploratory movements are possible for a given material, because we only know the limited number of EPs that participants use.

It is further worth noting that the materials in our study not only differed in how they were explored but also in how long they were explored. For example, the total time of exploration (obtained by adding the durations of all coded EPs) for slime and stress balls were longer than that of sand paper (average duration in seconds for slime: 82.2 s ± 8.3 SEM, stress balls: 78.7 s ± 3.5 SEM, sandpaper 51.5 s ± 6.1 SEM). Thus, participants kept moving their hands when making judgements about slime and stress balls, but preferred more sporadic contact with sandpaper. These differences can be explained by the different affective responses that these materials elicit: they tend to be negative for sandpaper, and are more positive for slime^14,15^. It is possible though that this affective component of tactile judgements not only contributes to the overall time that we spend exploring a material but that it also affects how the properties of a material are perceived. Further study is needed to investigate this possibility.

This study primarily investigated the changes of EP patterns as a function of the specific material properties. It is, however, also possible that participants not only use different EPs for different materials, but also use different EPs when rating different attributes of the same material^4,13^. Moreover, these two factors, i.e. the rating task and material properties could interactively influence which EPs are employed.

Haptic perception of materials is a heterogeneous process and this heterogeneity not only manifested itself in the results of a perceptual rating task but also in the EP patterns associated with specific perceptual dimensions. This work endorses the high dimensionality of the haptic representation of mechanical properties and of the material space itself by using a diverse set of stimuli and by letting observers interact with these freely. This underscores how much the theoretical constructs yielded by a study can be influenced by the choice of stimuli. Our work changes the conceptualization of EPs as being solely determined by the perceptual tasks; instead, they are also influenced by subtle changes in the material properties of objects.

## Methods

We used an adjective rating task, the semantic differential scaling method and principal component analysis to determine the perceptual dimensions of mechanical properties of real materials. Materials were selected to possess a variety of mechanical properties and to be compressible and deformable in different ways. Adjectives were selected to measure sensory aspects that cover a range of compressibility and compliance characteristics. We asked participants to haptically explore materials (no visual information was given) while rating them on a list of adjectives. During the experimental sessions, we also recorded videos of the hand motions of all participants. These videos were coded by raters to extract known and novel EPs. After this, we analyzed the differences in EP usage for materials scoring high on one of the obtained perceptual dimensions. Subsequently, we used EP patterns in a larger set of videos to train a support vector machine that would classify each material according to its perceptual dimensions.

### Participants

34 volunteers (17 females) took part in the experiment. The age range was 19–9 years (average age: 23.71 years). None of the participants had touch-related disabilities. A 2-point touch discrimination test on the right hand’s tips of the index finger was used prior to the experiment to ensure that the touch perception of the participant was in the normal range (individual thresholds 2– mm or better). The number of participants in the experiment was well above the typical number of 20 used in many previous similar experiments that studied perceptual dimensionality^16,17^. Before the study, participants gave written informed consent. The experiment was approved by the ethical review board of Bilkent University and in accordance with the Code of Ethics of the World Medical Association (Declaration of Helsinki). Participants were paid for their time after completing the experiment (30 Turkish Liras in total). The hand videos of 6 participants were excluded from the video analyses, because of recording errors (overwriting) or incomplete datasets.

### Video raters

Five raters coded the videos: three raters were students of the Justus-Liebig-University of Giessen, Germany; two of these coded the videos as part of their master thesis work; the third rater coded the videos as part of an undergraduate internship project. The two remaining raters were undergraduate Erasmus students from Konya University, Turkey. All raters participated in initial EP extraction and EP coding of 50 videos, one of these raters coded 100 videos used for SVM analyses.

### Stimuli

#### Materials

All nonrigid materials have in common that they can be dynamically deformed or changed in one way or another^12^. However, a dynamic deformation or change can occur in many ways: for example, running the fingers through sand alters its shape by displacing the individual grains, stroking fur compresses or bends the individual hairs, kneading a soft textile, a sponge or play dough deforms it through compression. All of these materials deform or change their shape - but they do so in idiosyncratic ways. In order to probe these different properties, we created a large set of 50 everyday deformable and non-deformable material items. For convenience, we organized these roughly into 5 categories: elastic materials, textiles, deformable materials, granulate materials and non-deformable materials (and as a control, to a small extend roughness), with 10 items per category. Table 3 provides a complete list of materials which we used.

**Table 3:** Materials that were used in the experiment, organized by salient material property.

	Materials
Elastic	Bandage, bouncy balls, juicy toy, lady’s stocking, latex, plush pillow, rubber bands, silicone, sponge, white rubber
Textile	Cotton, fur, leather, linen, mat, microfiber, polar, silk, velvet, wet cloth
Deformable	Aluminum foil, clay, hair gel, hand cream, paper, peeling cream, play dough, shaving cream, slime, wires
Granulate	Black pepper, chickpeas, flour, foam, pine cone, poppy seeds, red pepper, sand, sawdust, sugar
Non-soft	Brick, cardboard pieces, chalk, glass, hard plastic, metal nuts, sandpaper, stone, straw, wood block

#### Videos

Participants’ hand movements were recorded during the experiment by a Sony camera with a rate of 50 frames per s and HDMI resolution of 1280 p $\times$ 720 p. Video’s files were stored as XAVC SHD.

#### Adjective list

We started out with an initial set of 262 touch-related affective and sensory adjectives by adapting the haptic lexicon of Guest et al.^17^ to Turkish. We narrowed down the initial set by eliminating emotional and non-sensual adjectives and the ones that are too similar, and, finally, established a list of 31 adjectives that would describe mechanics (and roughness) related aspects of touch. Table 1 shows the list of adjectives used in our study along with their Turkish translation.

### Perceptual task

On a trial, participants reached out to a glass container that was hidden behind a black curtain occluder. The opening in the curtain occluder might have constrained the participants’ arms, but they were free to move their hands beyond the wrist while exploring. Their nose and ears were plugged and their task was to rate the degree to which each of the 31 adjectives would apply to each of the materials. Participants were specifically asked to rate the material, the word ‘object’ was not used during the instructions. While exploring one material item participants rated it on one adjective at a time (presented on a mini card) by saying out loud a number, that was noted down by the experimenter. The number was meant to express how much a given adjective was applicable/suitable to the material item (Semantic Differential Scale^18^); 1 indicating that the adjective was not applicable at all to the item and 7 indicating that the adjective very much applied. The orders of materials and adjectives were randomized. Participants had 3 s to respond to an adjective, and received a warning beep sound if this time interval passed. Each participant completed the experiment in one session that lasted approximately 90 min.

### Analysis

#### Consistency and exploratory factor analysis

In order to assess the consistency of the data across participants, we computed pairwise correlations of any two participants’ scores across all adjectives and materials, as well as Cronbach’s alpha between participants for individual adjectives. Because, participants’ judgments were highly consistent, we subsequently averaged across observers and conducted a covariance-based factor analysis with varimax rotation on the averages (for each adjective and material condition). We used the Keyser–Meyer–Olkin (KMO) test to assess the sampling adequacy of our variables and the model, as well as Bartlett’s test for sphericity in order to check for redundancies between variables before proceeding with a Factor Analysis.

#### Video coding

We first created a hand movement taxonomy from the recorded videos. This taxonomy included eight EPs, as shown in Fig. 1. These EPs were validated and used in subsequent analysis.

**EP extraction:** One rater watched 1400 videos (50 materials for 28 participants), and coded which parts of the hand were in contact with the materials during active exploration. This yielded an initial list of 8 exploratory hand movement schemes. Movements that mainly serve to bring a given material into the ‘right’ global position, such as dropping or picking up, were not considered as EPs, nor were ‘maintenance’ movements, like cleaning material parts off the hands (e.g. sand, by shaking). During this refinement stage, we removed hand movements from analysis that did not serve any purpose for perception (such as cleaning movements) and also decided to pick one EP per time segment, for instance, if four digits were used to hold a material, such as a velvet cloth, and the index finger is used to stroke its surface, we decided to code stroking alone.

In a second refinement step, we selected a total of 5 videos: black pepper, sponge, slime, stone, plush pillow. The authors watched these videos together with all 5 raters and reiteratively discussed the observed events. Through these discussions, we identified eight specific hand movements that were coded the same by all raters and that occurred frequently for many materials: pressing, rubbing, stroking, rotating, stirring, pulling, running through, and tapping. Examples of each of these EPs are shown in Fig. 1. This second step also trained raters for subsequent event coding, EP taxonomy, and software usage.

**EP coding:** Using the results of the factor analysis described in the Exploratory Factor Analysis Results, we selected the two most representative materials for each perceptual dimension (5 in total, Table 1), for the coding of video sequences for EPs. Specifically, we chose the video sequences of materials that yielded high factor scores for a particular perceptual dimension, while having the lowest sum of factor scores in the remaining perceptual dimensions. Using these criteria, we selected stress balls and sponge (which load high on compliance), slime and hand cream (loading high on viscosity), velvet and fur (loading high on surface softness), sand and poppy seeds (loading high on granularity), and as a control dimension, scourer and sandpaper (which load high on roughness).

**Figure 4:**
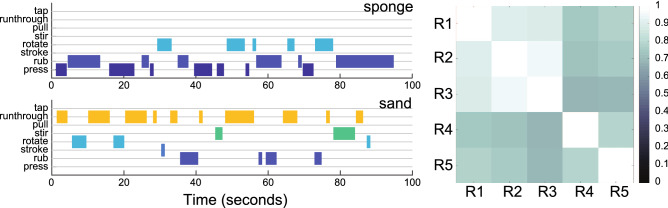
Left: Timeline output from BORIS shows two example observations for sponge (top) and sand videos (bottom, of one rater and 1 participant). For sponge, the observation starts with pressing and then rubbing, where these two EPs are intermittently coded at the beginning (first 30 s) of the video, then rotating is also included. Similarly, for sand, the rater codes running through and rotation; then stroking, rubbing, and stirring. For EP frequency analysis, the durations of a given EP are added and divided by the total duration of all EPs. Right: Shown are the correlation coefficients between raters for 50 video clips, where each square represents the average of correlation coefficients of pairwise correlations of 50 $\times$ 8 arrays of EP frequencies per rater. Lighter colors indicate higher correlation coefficients.

For each perceptual dimension, we selected 10 videos (5 per exemplary material). Videos were randomly drawn from the entire participant pool, allowing to treat observations in the different levels of perceptual dimension as being independent. Each of the five raters coded the 50 videos. They were asked to focus on the parts of the hand that are in contact with the material. Each video clip would start by depicting the grasping and lifting of the material (or pieces of it). Raters were asked to start event coding for EPs after this initial period and to focus specifically on the subsequent procedures. The maximum duration of a video clip was 2 min. During time periods where multiple fingers are in contact with the object, the moving hand parts define the EP: for example, when two fingers hold the material, while the index finger starts rubbing the surface, it was coded as rubbing. If multiple actions were observable in different parts of the hand, raters coded only the most prominent EP. A supplementary movie showing each EP recorded from various participants can be found at: https://vimeo.com/393420896. Raters marked each EP’s start and end times using an event logging software: Behavioral Observation Research Interactive Software (BORIS^19^, illustrated in Fig. 4, left). EP coding took a minimum of 20 hours per rater—excluding the training period. All further frequency and classification analyses were performed in MATLAB (v2018b, The Mathworks, USA) and statistical tests were done with IBM SPSS Statistics for Windows, version 23.0 (IBM Corp., Armonk, N.Y., USA).

**Inter-rater reliability:** We first calculated each EP’s frequency per video by taking the ratio of the duration of one EP to the sum of total EP durations coded for that video. Consequently, the relative frequencies of all 8 EPs add up to 1 (for each video). We use the term frequency because the EP during a prolonged duration period would be executed several times. To measure the inter-rater reliability for the five raters, we calculated Pearson correlation coefficients of EP relative frequencies for corresponding videos coded by different raters and report averaged R values (Fig. 4, right). This allowed us to assess whether our EPs were well-defined and sufficient to describe the hand movements in the video material.

**Frequency analysis of EPs:** After establishing inter-rater reliability and averaging EP data across 5 raters we investigated the average relative frequency of the novel set of EPs in the subset of 10 representative materials (5 participants each) described in the Stimuli, Materials section. Our conjecture was that participants would have different exploration strategies for different perceptual dimensions. We compared patterns of 8 EP frequencies as dependent variables in a one-way MANOVA with perceptual dimensions (compliance, viscosity, surface softness, granularity, and roughness as a control) as a fixed factor. EP frequencies were averaged across raters. For each perceptual dimension 5 $\times$ 2 = 10 observations (patterns of EP frequencies) entered the MANOVA given by 5 participants exploring each of the 2 materials belonging to that dimension. We report Wilk’s lambda for the EP frequency in Results section.

#### Classification

We tested the performance of a support vector machine with a Gaussian kernel predicting the perceptual dimension of a material given the EP frequency data. Our data set consisted of videos from 10 participants (5 that were rated before and 5 additional) exploring 10 materials (see above). We fit a multiclass model (5 dimensions, chance level 20%) for a support vector machine (Statistics and Machine Learning Toolbox, Matlab, The MathWorks, USA) to predict a material’s perceptual dimension given its EP frequency pattern. We then perform a 10-fold cross validation where the model randomly trains with 90 videos and tests on 10 videos. After repeating this 10 times, each time randomly partitioning the data set 10–9039observations) was similar to the test set (remaining 10 observations); for the next iteration the training set would be a different group of 90 observations. We report mean posterior probabilities for each perceptual dimension.

## Data Availability

The datasets generated and/or analysed during the current study are available in the Zenodo repository, under the name: Dicle N Dövencioǧlu, Seyhun Ustun, Katja Doerschner, and Knut Drewing. (2020). From silk to sand: Multiple dimensions of perceived softness [Data set]. Zenodo. https://doi.org/10.5281/zenodo.6912671.
